# NR4A1 Deletion in Marginal Zone B Cells Exacerbates Atherosclerosis in Mice—Brief Report

**DOI:** 10.1161/ATVBAHA.120.314607

**Published:** 2020-09-10

**Authors:** Meritxell Nus, Gemma Basatemur, Maria Galan, Laia Cros-Brunsó, Tian X. Zhao, Leanne Masters, James Harrison, Nichola Figg, Dimitrios Tsiantoulas, Frederic Geissmann, Christoph J. Binder, Andrew P. Sage, Ziad Mallat

**Affiliations:** 1 Division of Cardiovascular Medicine, Department of Medicine, University of Cambridge, United Kingdom (M.N., G.B., L.C.-B., T.X.Z., L.M., J.H., N.F., A.P.S., Z.M.).; 2 CIBER de Enfermedades Cardiovasculares, Spain (M.N., M.G.).; 3 Biomedical Research Institute, Hospital de la Santa Creu i Sant Pau, Barcelona, Spain (M.G.).; 4 Department of Laboratory Medicine, Medical University of Vienna, Austria (D.T., C.J.B.).; 5 Memorial Sloan Kettering Cancer Centre, New York (F.G.).; 6 Institut National de la Santé et de la Recherche Médicale, Unit 970, Paris Cardiovascular Research Center, France (Z.M.).

**Keywords:** animals, atherosclerosis, cholesterol, diet, Western, mice

## Abstract

NR4A orphan receptors have been well studied in vascular and myeloid cells where they play important roles in the regulation of inflammation in atherosclerosis. NR4A1 (nerve growth factor IB) is among the most highly induced transcription factors in B cells following BCR (B-cell receptor) stimulation. Given that B cells substantially contribute to the development of atherosclerosis, we examined whether NR4A1 regulates B-cell function during atherogenesis.

**Approach and Results:**

We found that feeding *Ldlr*^−/−^ mice a Western diet substantially increased *Nr4a1* expression in marginal zone B (MZB) cells compared with follicular B cells. We then generated *Ldlr*^−/−^ mice with complete B- or specific MZB-cell deletion of *Nr4a1*. Complete B-cell deletion of *Nr4a1* led to increased atherosclerosis, which was accompanied by increased T follicular helper cell–germinal center axis response, as well as increased serum total cholesterol and triglycerides levels. Interestingly, specific MZB-cell deletion of *Nr4a1* increased atherosclerosis in association with an increased T follicular helper–germinal center response but without any impact on serum cholesterol or triglyceride levels. *Nr4a1*^−/−^ MZB cells showed decreased PDL1 (programmed death ligand-1) expression, which may have contributed to the enhanced T follicular helper response.

**Conclusions:**

Our findings reveal a previously unsuspected role for NR4A1 in the atheroprotective role of MZB cells.

**Graphic Abstract:**

A graphic abstract is available for this article.

## Abstract 2

Supplemental Digital Content is available in the text.

**Highlights**

- NR4A1 is a new target that modulates B-cell functions in atherosclerosis.
- *Nr4a1* deletion in B2 cells increases atherosclerosis and is associated with increased Tfh-GC response and circulating lipid levels.
- NR4A1 expression in MZB cells regulates PDL1 expression, limiting the Tfh-GC response and protecting from atherosclerosis.

Atherosclerosis is a chronic inflammatory disease involving interactions between vascular, circulating, and immune cells. B cells play an important role in the chronic immunoinflammatory response, producing antibodies and regulating T-cell and natural killer–cell activation. The role of B cells in atherosclerosis is complex, with atherogenic and protective roles assigned for distinct B-cell subsets.^1^ B2 cells comprise the phenotypically distinct follicular B (FOB) cells of the spleen and the lymph nodes and the marginal zone B (MZB) cell population of the spleen. While depletion of all B2 cells reduces atherosclerosis,^2–4^ we have recently discovered that selective depletion of MZB cells promotes atherosclerosis.^5^ In response to a high-fat/high-cholesterol (HF/HC) diet, MZB cells activate an atheroprotective programme, limiting T follicular helper (Tfh) cell motility in a PDL1 (programmed death ligand-1)-dependent manner.^5^

NR4A1 (nerve growth factor IB; also called Nur77) is a member of the NR4A orphan nuclear receptor subfamily. NR4A1 has been involved in negative selection of thymocytes, differentiation of regulatory T cells, and development of Ly6C^−^ monocytes.^6^ However, little is known about its role in B cells besides the fact that it is rapidly induced upon BCR (B-cell receptor) signaling activation^7^ and that it may regulate the survival of self-reactive B cells.^8^ NR4A1 also plays important but complex roles in atherosclerosis, at least in part, due to its impact on vascular cell,^9^ monocyte,^6,10,11^ and T-cell biology.^6,12^ We have now addressed the role of NR4A1 specifically in B cells in the development of atherosclerosis.

## Materials and Methods

The data that support the findings of this study are available from the corresponding author upon reasonable request.

### Animals

All experiments were approved by the Home Office, United Kingdom, and were performed under Personal Project Licence 80/2426. All mice were on *C57Bl/6J* background. *Cd79a*^*Cre/+*^, *µMT*, *Nr4a1*^−/−^, and Ldlr^–/–^ mice were originally from The Jackson Laboratory. *Rbpjk*^*flox/flox*^ mice were kindly provided by Tasuku Honjo.^13^ To generate an atherosclerotic mouse model that lacks MZB cells, *Cd79a*^*Cre/+*^; *Rbpjk*^*flox/flox*^ were crossed with *Ldlr*^–/–^ to generate *Ldlr*^−/−^*; Cd79a*^*Cre/+*^*; RBP*^*flox/flox*^ mice. For atherosclerosis experiments, we generated 2 different mice models. For deletion of *Nr4a1* in all B cells, 6- to 8-week-old *Ldlr*^–/–^ mice were lethally irradiated (two 5.5 Gy fractioned doses) and injected with a mixed bone marrow (BM) chimera containing 80% *uMT* plus either 20% WT (wild type) C57Bl/6J (to reconstitute with WT B cells) or 20% *Nr4a1*^−/−^ (to reconstitute with *Nr4a1*^−/−^ B cells). We only used male mice to limit the number of animals used in our experiments. In subsequent experiments, we used both males and females. For deletion of *Nr4a1* selectively in MZB cells, we developed a partial irradiation model in atherosclerotic mice lacking MZB cells. Recipient *Ldlr*^−/−^*; Cd79a*^*Cre/+*^*; RBP*^*flox/flox*^ male and female mice were partially irradiated (1 single dose of 4 Gy) and injected intravenously with BM from *Nr4a1*^−/−^ or WT C57Bl/6J male and female mice, respectively, which we expected to result in reconstitution of MZB cell compartment with cells coming from the donors (*Nr4a1*^−/−^ or WT), while the rest of immune cells (including FOB, T, myeloid, etc) would be mostly from the recipient. To validate this partial irradiation model, we used the CD45 (cluster of differentiation 45) congenic lineage tracing system. We exposed recipient CD45.2+; CD45.1− male and female mice lacking MZB cells (*LDLr*^−/−^*; Cd79a Cre/*^+^; *RBP*^*flox/flox*^) to different doses of irradiation (from 4 to 7 Gy), then injected CD45.2−; CD45.1+ male and female BM donor cells, respectively, to allow us to track the reconstitution of B-cell subsets using flow cytometry. A single dose of 4 Gy resulted in reconstitution of the majority of MZB cells (>99%) with donor CD45.2−; CD45.1+ cells, while the majority of the FOB cells and the rest of the BM-derived cells (including other lymphocytes [FSC^low^] and myeloid [FSC^hi^] cells; 90%) were from the CD45.2+; CD45.1− recipient (Figure I in the Data Supplement).

For atherosclerotic experiments, all recipient mice were randomly assigned to different experimental groups based on their weight at the beginning of the experiment. After 6 weeks of recovery, mice were started on HF/HC (21% fat, 0.15% cholesterol) for 8 weeks.

### Extent and Composition of Atherosclerotic Lesions

The lesions in the root of the aorta beneath all 3-valve leaflets were analyzed. To measure atherosclerotic plaque size, we stained paraffin-embeded sections with Masson trichrome. To characterize atherosclerotic lesion composition, we stained sections for macrophages (Mac-3 [lysosome-associated membrane protein 2], 1:200, clone M3/84; Santa Cruz), T cells (polyclonal CD3, 1:100; Dako), and neutrophils (Ly6G [lymphocyte antigen 6 complex locus G6D], 1:100, clone 1AB; BD). We performed each staining on a single slide (3 sections for each staining) from approximately the same level of the aortic root for all experimental and control mice. Computer-assisted analysis (Image-J) was used to determine lesion size and areas or cell counts for specific cell types. All slides for each experiment were analyzed by the same blinded observer to minimize variations. All measurements were performed by manual selection of stained pixel thresholds and presented as percent positive (stained) area relative to the entire intimal area. CD4^+^ T and Ly6G^+^ cells were counted, and the data are presented as the number of T cells per total number of cells or as the total number of cells per section, respectively, in atherosclerotic plaques and adventitia. Representative images of immunofluorescence staining are included in Figure II in the Data Supplement. Whole aortas were prepared en face for Oil Red O staining to measure lipid deposition and quantification of lesion size using J-Microvision.

### Flow Cytometry

Single-cell suspensions of homogenized spleen and inguinal lymph nodes were stained with fluorophore-conjugated antibodies and analyzed using an LSRII Fortessa (Becton, Dickinson and Company) or FACSCantoII (BD) flow cytometer as described previously.^5^ Representative plots of gating strategies can be found in Figure II in the Data Supplement.

### Determination of Circulating Antibodies

Specific antibody titers to given antigens in plasma were determined by chemiluminescent ELISA as described previously.^14,15^

### Determination of Serum Lipid Levels

Total cholesterol, triglycerides, and HDL-C were measured by an enzymatic method in a Siemens Dimensions RxL analyzer.

### Purification of MZB and FOB

Single-cell suspensions were prepared from spleens by dissociation through 70-µm filters using a syringe plunger. For MZB and FOB cell purification, B cell–enriched populations were separated by Automacs using a B-cell purification kit (Miltenyi Biotech), and B cells were stained for 15 minutes at 4°C with anti-CD23-phycoerythrine and anti-CD21-fluorescein isothiocyanate. After washing and staining with 7-AAD for cell viability, MZB (CD21^hi^CD23^low^) and FOB (CD23^hi^CD21^low^) were sorted using an AriaIII Cell, Influx, or FACSJazz sorter (BD). Purity of both populations was higher than 95%.

### BCR Stimulation

Purified sorted MZB and FOB from WT or *Nr4a1*^−/−^ mice were stimulated for 30 minutes, 3 hours, or 6 hours with F(ab)2 fragment of the anti-IgM (eBioscience; 10 ug/mL).

### Quantitative Real-Time Polymerase Chain Reaction

For gene expression analysis, RNA from sorted MZB and FOB cells was isolated using RNAeasy mini kit (Qiagen). qRT-PCR was performed using Quantitect whole transcriptome kit. qRT-PCR was performed with SYBR-green primers. Primer sequences are listed in Table I in the Data Supplement.

### Statistical Analysis

Values are expressed as means±SEM. Based on pilot experiments, we found that MZB deficiency increases lesion size by 2-fold. Use of the equation for Student *t* test for the difference of 2 means gives us an n=6 to detect a 50% increase in lesion size with α=0.05 and 90% power. Where data sets passed normality tests, differences between values were examined using parametric 2-tailed unpaired Student *t* test, other data sets by using nonparametric Mann-Whitney *U* test; and were considered significant at *P*<0.05 for the B-cell model where only males were used. In the partial irradiation model, we used both males and females and performed 2-way ANOVA to determine comparisons between genotypes. When sex did not have a significant effect on the results, we pooled the male and female data. When sex had a significant effect, data were not pooled, and Bonferroni post hoc analysis was performed to find differences between groups.

## Results

### NR4A1 Deletion in B Cells Exacerbates Atherosclerosis

**Figure 1.**
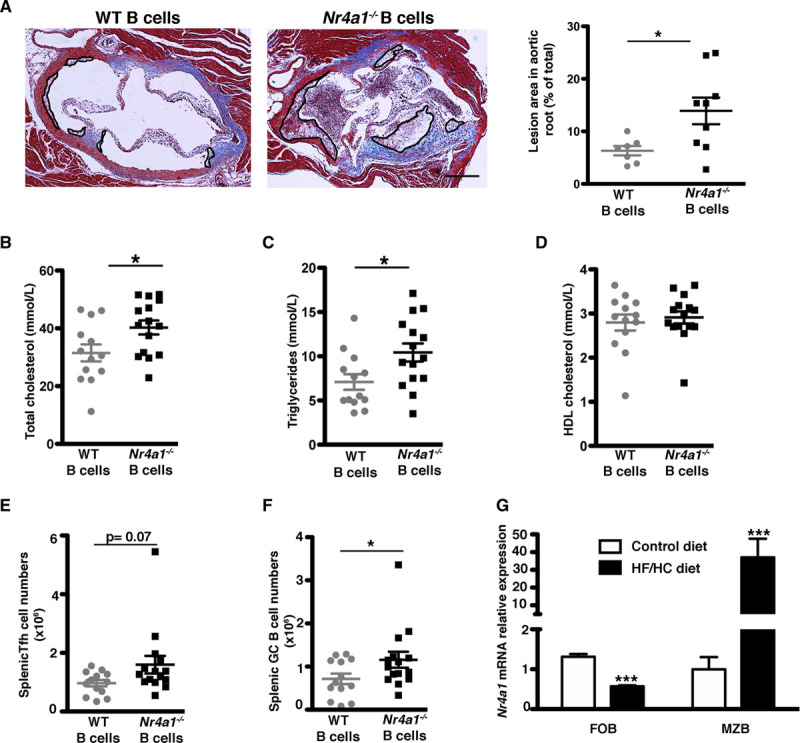
***Nr4a1* (nerve growth factor IB) deletion in B cells increases atherosclerosis development.** *Ldlr*^−/−^ mice were transplanted with a mixed chimera containing 80% *µMT* +20% WT (wild type; for reconstitution with WT B cells) or 20% *Nr4a1*^−/−^ (for reconstitution with *Nr4a1*^−/−^ B cells) BM and fed a high-fat/high-cholesterol (HF/HC) diet for 8 wk (**A–F**). **A**, Representative Masson trichrome staining and quantification of atherosclerotic plaques in aortic roots (each symbol represents one mouse, and the horizontal bars are group mean±SEM with n=7 and n=9, respectively, in each group). Original magnification, ×10. Scale bars=500 μm. **B–D**, Total plasma cholesterol, triglycerides, and HDL (high-density lipoprotein) cholesterol levels. **E** and **F**, Total numbers of T follicular helper (Tfh) cells (CD3^+^ CD4^+^ CD44^+^ CD62L^−^ ICOS^+^ CXCR5^+^ PD1^+^) and germinal center (GC) B cells (B220^+^ CD19^+^ CD95^+^ GL7^+^; n=13–15 in each group). **G**, *Nr4a1* relative expression in sorted marginal zone B (MZB) and follicular B (FOB) cells from *Ldlr*^−/−^ mice fed a standard laboratory or HF/HC diet for 8 wk (n=3–4 in each group). For **A–G**, 2-tailed unpaired Student *t* test or 2-way ANOVA followed by Bonferroni post hoc analysis, *P<0.05 and ***P<0.001. CD indicates cluster of differentiation; CXCR5, C-X-C chemokyne receptor-5; ICOS, inducible T-cell coestimulator; and PD1, programmed cell death protein 1.

We reconstituted lethally irradiated *Ldlr*^−/−^ male mice with a mixed BM containing 80% of BM cells from *µMT* male mice (which cannot produce B cells) supplemented with either 20% BM from WT C57Bl/6J mice (referred to as the WT B-cell group) or 20% of BM cells from *Nr4a1*^−/−^ mice (referred to as the *Nr4a1*^−/−^ B-cell group). MZB and FOB cells were reconstituted equally in both groups (Figure III in the Data Supplement). After recovery, mice were put on a high-fat/high-cholesterol diet for 8 weeks. Specific *Nr4a1* deletion in B cells markedly accelerated the development of atherosclerosis in the aortic sinus (Figure 1A) and the aortic arch (Figure III in the Data Supplement). Serum levels of total cholesterol and triglycerides were also significantly increased in the *Nr4a1*^−/−^ B-cell group (Figure 1B through 1D), which could account, at least in part, for the increased atherosclerosis seen in these mice. Analysis of plaque composition showed a trend toward increased %necrotic core area but a similar percentage of macrophages and CD3^+^ T cells in lesions of *Nr4a1*^−/−^ compared with WT B-cell mice (Figure III in the Data Supplement). In the adventitia, we found similar percentages of CD3^+^ T cells and a trend toward increased percentages of neutrophils (Ly6G^+^) in the *Nr4a1*^−/−^ compared with WT B-cell mice (Figure III in the Data Supplement). We did not find any difference in blood immune cell subsets (including B cells, T cells, neutrophils, and monocytes) between both groups (Figure III in the Data Supplement).

We then characterized the innate and the adaptive immune responses in the spleen. Splenic dendritic cell, monocyte, and neutrophil levels were similar between the 2 groups of mice (Figure III in the Data Supplement). *Nr4a1*^−/−^ B-cell mice showed a trend toward increased Tfh cell numbers (*P*=0.07) and had significantly increased germinal center (GC) B cells (*P*<0.05) compared with mice with WT B cells (Figure 1E and 1F). All other T-cell subsets including T effector memory, T regulatory cells, and T follicular regulatory cells were not significantly altered between the two groups (Figure IV in the Data Supplement).

We have previously demonstrated that B2 cells play different roles in atherosclerosis, with MZB cells being atheroprotective through limitation of Tfh cell formation (MZB cell deletion leads to increased Tfh)^5^—a phenotype that is similar to the present phenotype observed for B cell–specific depletion of *Nr4a1*^−/−^. Furthermore, expression of *Nr4a1* was significantly upregulated in purified MZB cells but downregulated in FOB cells from *LDLr*^−/−^ mice consuming a high-fat/high-cholesterol diet, in comparison with mice fed a standard laboratory diet (Figure 1G). Therefore, we addressed the role of NR4A1 specifically in MZB cells during the development of atherosclerosis.

### NR4A1 Deletion Selectively in MZB Cells Exacerbates Atherosclerosis

To study the role of Nr4a1 in MZB cells and its impact on atherosclerosis, we generated an atherosclerotic mouse model in which *Nr4a1*^−/−^ was specifically deleted in MZB cells. For this aim, we generated *Ldlr*^−/−^ mice on *Cd79a*^*Cre/+*^*; RBP*^*flox/flox*^ background (mice that do not have MZB cells, as described previously^5^). After partial irradiation (4 Gy), *Ldlr*^−/−^*; Cd79a*^*Cre/+*^*; RBP*^*flox/flox*^ male and female mice were injected with WT or *Nr4a1*^−/−^ BM from males and females, respectively, resulting in mice reconstituted with WT MZB cells (the WT MZB cell group) or with MZB cells in which *Nr4a1* has been deleted (the *Nr4a1*^−/−^ MZB cell group; see Materials and Methods section for a more detailed explanation of the model and Figure I in the Data Supplement). Both groups had the same numbers of MZB and FOB cells after reconstitution (Figure I in the Data Supplement).

**Figure 2.**
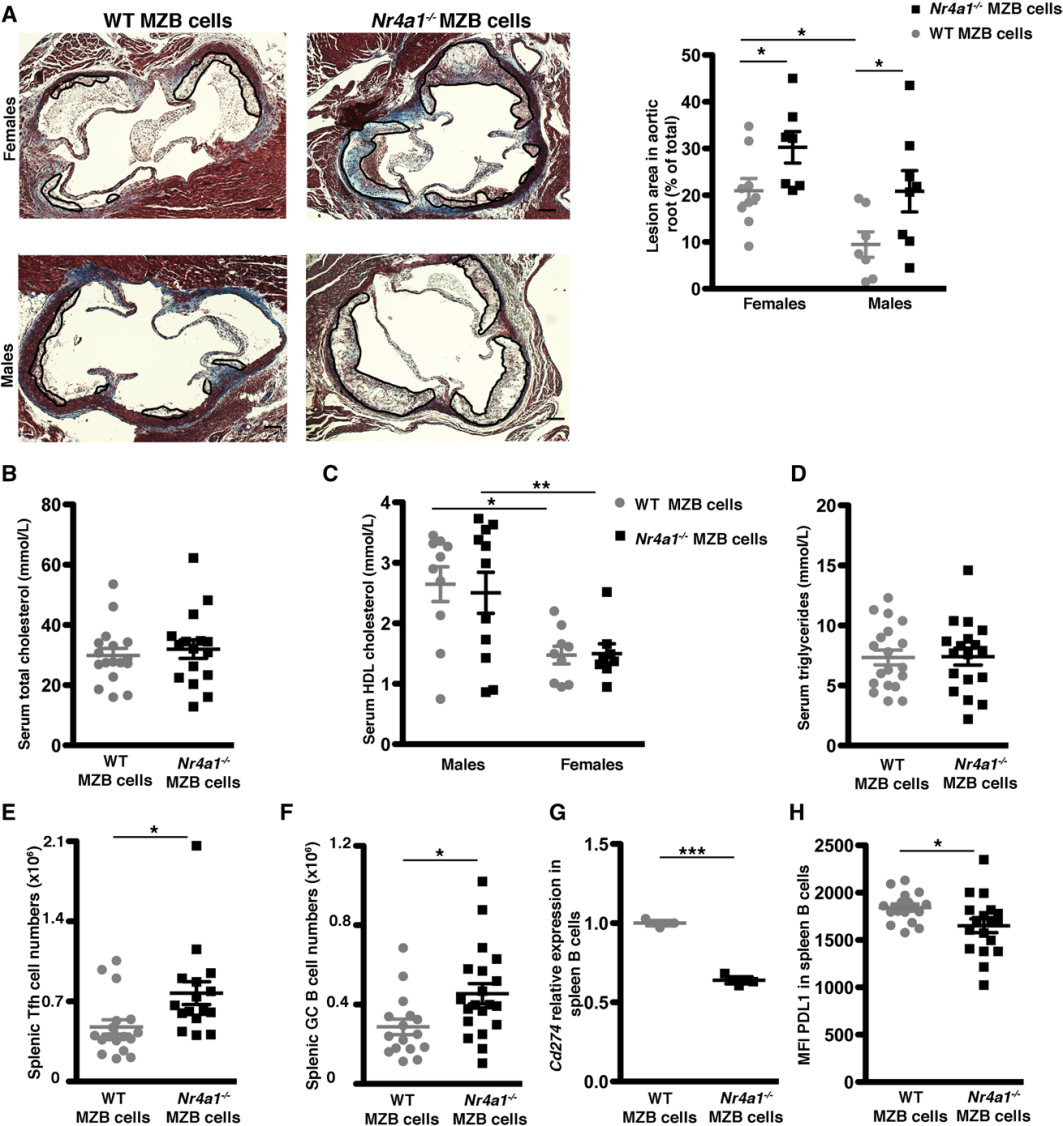
***Nr4a1* (nerve growth factor IB) deletion in marginal zone B (MZB) cells increases atherosclerosis development.** *Ldlr*^−/−^*; Cd79a*^*Cre/+*^*; Rbpjk*^*flox/flox*^ were partially irradiated (Materials and Methods in the Data Supplement) and transplanted with WT (wild type; for reconstitution with WT MZB cells) or *Nr4a1*^−/−^ (for reconstitution with *Nr4a1*^−/−^ MZB cells) BM and fed a high-fat/high-cholesterol diet for 8 wk (**A–H**). **A**, Representative Masson trichrome staining and quantification of atherosclerotic plaques in aortic roots (each symbol represents one mouse, and horizontal bars are group mean±SEM with n=16–18 in each group). Original magnification, ×10. Scale bars=500 μm. **B–D**, Total plasma cholesterol, HDL (high-density lipoprotein) cholesterol, and triglycerides levels. **E** and **F**, Total numbers of T follicular helper (Tfh) and germinal center (GC) B cells. *Cd274* (*Pdl1* [programmed death ligand-1] gene) expression, quantified by qRT-PCR (n=5 in each group) in sorted MZB cells (**G**) and PDL1 protein expression, quantified by flow cytometry in splenic MZB cells (**H**; n=16–18 in each group). For **A–G**, 2-tailed unpaired Student *t* test or 2-way ANOVA followed by Bonferroni post hoc analysis, *P<0.05 and **P<0.01. MFI indicates mean fluorescence intensity; and PDL1, programmed cell death ligand-1.

After 8 weeks on a high-fat/high-cholesterol diet, deletion of *Nr4a1* specifically in MZB cells led to increased development of atherosclerosis in the aortic sinus (Figure 2A) and increased lipid deposition in the aortic arches (Figure V in the Data Supplement), without altering serum levels of total cholesterol, HDL-C, or triglycerides, compared with mice with WT MZB cells (Figure 2B through 2D). Females had increased atherosclerotic plaques and decreased HDL-C levels than males—a phenotype that has been reported previously in *LDLr*^−/−^ mice.^16^ Analysis of plaque composition showed a trend toward increased %necrotic core area in the *Nr4a1*^−/−^ MZB cell group while there were no differences in percentages of Mac-3^+^ macrophages and CD3^+^ T cells between both groups (Figure V in the Data Supplement). No significant differences were found in the percentages of CD3+ T cells and neutrophils in the adventitia between the 2 groups (Figure V in the Data Supplement).

Interestingly, *Nr4a1*^−/−^ MZB-cell mice had significantly increased splenic Tfh and GC B-cell numbers (Figure 2E and 2F) compared with WT MZB-cell mice, without detectable changes in serum antibody titers against modified LDL (low-density lipoprotein; Figure V in the Data Supplement). Despite our limitations to comprehensively assess the antibody response, we hypothesize that other Tfh cell functions may account for their atherogenic role, as discussed previously.^5^ *Nr4a1*^−/−^ MZB cell group also had significantly increased T regulatory cells and T follicular regulatory CD25^pos^ cells, while T effector memory, T follicular regulatory CD25^neg^ cells, dendritic cells, neutrophils, and monocytes were not significantly altered (Figure VI in the Data Supplement).

### NR4A1 Regulates PDL1 Expression

We found that MZB cells from the *Nr4a1*^−/−^ MZB group expressed lower levels of the PDL1 encoding gene (*Cd274*) and PDL1 protein (Figure 2G and 2H) compared with those from the WT MZB group, which could, at least in part, explain why the *Nr4a1*^−/−^ MZB cell group also showed increased Tfh cells.^5^

To further examine the link between NR4A1 and PDL1, we performed in vitro experiments using sorted MZB cells from total WT and *Nr4a1*^−/−^ mice. MZB cells from *Nr4a1*^−/−^ mice expressed lower levels of PDL1 than WT MZB cells at baseline (Figure VII in the Data Supplement). As described previously, *Nr4a1*^17^ and *Cd274*^5^ significantly increased upon BCR activation (Figure VII in the Data Supplement). BCR-induced pBTK (Bruton tyrosine kinase) was not altered in the absence of NR4A1 (Figure VII in the Data Supplement). BCR-induced *Cd274* gene expression was significantly downregulated in the absence of NR4A1 (Figure VII in the Data Supplement). However, BCR-mediated regulation of MZB cell surface PDL1 protein expression was not altered in the absence of NR4A1 (Figure VII in the Data Supplement). These data suggest that the impact of NR4A1 deletion on PDL1 expression in vivo was independent of BCR activation.

## Discussion

To the best of our knowledge, this is the first experimental study addressing the role of NR4A1 in B cells during atherosclerosis. Our data show that NR4A1 expression in MZB cells regulates PDL1 expression, limiting the Tfh-GC response and protecting from atherosclerosis. A recent work performed in a cancer line has shown that NR4A1 binds to the GC motif of the PDL1 promoter and regulates its gene expression,^18^ further supporting the possibility of a direct regulation of PDL1 expression by NR4A1 in MZB cells. Previous studies suggested that PDL1 expression in antigen-presenting cells might limit proatherogenic immune responses.^19^ Whether NR4A1 also regulates PDL1 expression in immune cells, other than MZB cells, is worth of investigation.

Interestingly, NR4A1 may regulate PDL1 in a BCR-independent manner, opening up an opportunity to modulate NR4A1-dependent pathways in B cells without affecting the BCR signaling pathway. Furthermore, human MZB-like cells express high levels of NR4A1^20^ and PDL1,^5^ and, therefore, we speculate that NR4A1 may have a similar role in human B cells, although further studies are required to determine whether this is indeed the case. The potential use of Bis-Indole-NR4A1 antagonists as anticancer treatments is starting to emerge,^18,21^ and, therefore, we would advise to take into consideration their effect on B cells to avoid or monitor potential undesirable cardiovascular side effects associated to anticancer treatments.

NR4A1 has previously been reported to play a very important role in B-cell survival.^8,22^ Nevertheless, in both of our B-cell models of *Nr4a1* deletion, we did not find any difference in B-cell numbers, making it unlikely that NR4A1 regulates B-cell survival during atherosclerosis. This is in agreement with a recent finding that NR4A1 regulates B-cell survival in a T cell–independent response but not in a T cell–dependent response.^23^ In our models, we could not study the potential contribution of NR4A1 in B1 cells because these cells do not reconstitute well after irradiation, and, therefore, further experiments are needed to elucidate the role of NR4A1 in B1 cells during atherosclerosis.

Surprisingly, NR4A1 deletion in all B2 cells not only regulates the Tfh-GC response but also the circulating lipid levels. Thus, B2 cells other than MZB cells could be responsible for this effect on lipid parameters. B cells express several receptors, such as LDLR (LDL receptor), CD1d, and scavenger receptors, that could recognize and remove lipids.^24^ Moreover, previous studies modulating B cells have shown changes in cholesterol levels,^25,26^ but none of the previous atherosclerotic models targeting NRA1 in monocytes have shown a significant effect on lipid levels.^10,11^ Thus, the specific role of B cells in lipid metabolism is yet to be clarified, and further experiments are required.

Overall, our data demonstrate that NR4A1 in B cells is an attractive target to modulate the development of atherosclerotic disease.

## Acknowledgments

We acknowledge Prof Anna Petrunkina, Natalia Savinykh, Nika Romashova, and Simon McCallum, in the Phenotyping Hub of the Department of Medicine (University of Cambridge), for their help in flow cytometry and sorting and Maria Ozsvar Kozma (Department of Laboratory Medicine, Medical University of Vienna, Austria) for help in antibody measurements. We also acknowledge Keith Burling and all the Core Biomedical Assay Laboratory in Addenbrooke Hospital for lipid measurements.

## Sources of Funding

This work was supported by the British Heart Foundation (BHF) Project grant PG/17/73/33251 to M. Nus and Z. Mallat and by the European Union grant RG71687 to Z. Mallat. We were also supported by the Cambridge BHF Centre of Research Excellence and NIHR Cambridge Biomedical Research Centre.

## Disclosures

None.

## Supplementary Material



## References

[1] Nus M Tsiantoulas D Mallat Z Plan B (-cell) in atherosclerosis. Eur J Pharmacol. 2017; 816:76–81. doi: 10.1016/j.ejphar.2017.09.002 28882560 10.1016/j.ejphar.2017.09.002

[2] Ait-Oufella H Herbin O Bouaziz JD Binder CJ Uyttenhove C Laurans L Taleb S Van Vré E Esposito B Vilar J B cell depletion reduces the development of atherosclerosis in mice. J Exp Med. 2010; 207:1579–1587. doi: 10.1084/jem.20100155 20603314 10.1084/jem.20100155 PMC2916123

[3] Kyaw T Cui P Tay C Kanellakis P Hosseini H Liu E Rolink AG Tipping P Bobik A Toh BH BAFF receptor mAb treatment ameliorates development and progression of atherosclerosis in hyperlipidemic ApoE(-/-) mice. PLoS One. 2013; 8:e60430 doi: 10.1371/journal.pone.0060430 23560095 10.1371/journal.pone.0060430 PMC3616162

[4] Sage AP Tsiantoulas D Baker L Harrison J Masters L Murphy D Loinard C Binder CJ Mallat Z BAFF receptor deficiency reduces the development of atherosclerosis in mice–brief report. Arterioscler Thromb Vasc Biol. 2012; 32:1573–1576. doi: 10.1161/ATVBAHA.111.244731 22426131 10.1161/ATVBAHA.111.244731

[5] Nus M Sage AP Lu Y Masters L Lam BYH Newland S Weller S Tsiantoulas D Raffort J Marcus D Marginal zone B cells control the response of follicular helper T cells to a high-cholesterol diet. Nat Med. 2017; 23:601–610. doi: 10.1038/nm.4315 28414328 10.1038/nm.4315

[6] Hamers AA Hanna RN Nowyhed H Hedrick CC de Vries CJ NR4A nuclear receptors in immunity and atherosclerosis. Curr Opin Lipidol. 2013; 24:381–385. doi: 10.1097/MOL.0b013e3283643eac 24005216 10.1097/MOL.0b013e3283643eac PMC4709022

[7] Zikherman J Parameswaran R Weiss A Endogenous antigen tunes the responsiveness of naive B cells but not T cells. Nature. 2012; 489:160–164. doi: 10.1038/nature11311 22902503 10.1038/nature11311 PMC3438375

[8] Tan C Mueller JL Noviski M Huizar J Lau D Dubinin A Molofsky A Wilson PC Zikherman J Nur77 links chronic antigen stimulation to B cell tolerance by restricting the survival of self-reactive B cells in the periphery. J Immunol. 2019; 202:2907–2923. doi: 10.4049/jimmunol.1801565 30962292 10.4049/jimmunol.1801565 PMC6504591

[9] You B Jiang YY Chen S Yan G Sun J The orphan nuclear receptor Nur77 suppresses endothelial cell activation through induction of IkappaBalpha expression. Circ Res. 2009; 104:742–749. doi: 10.1161/CIRCRESAHA.108.192286 19213954 10.1161/CIRCRESAHA.108.192286

[10] Hamers AA Vos M Rassam F Marinković G Marincovic G Kurakula K van Gorp PJ de Winther MP Gijbels MJ de Waard V Bone marrow-specific deficiency of nuclear receptor Nur77 enhances atherosclerosis. Circ Res. 2012; 110:428–438. doi: 10.1161/CIRCRESAHA.111.260760 22194623 10.1161/CIRCRESAHA.111.260760

[11] Chao LC Soto E Hong C Ito A Pei L Chawla A Conneely OM Tangirala RK Evans RM Tontonoz P Bone marrow NR4A expression is not a dominant factor in the development of atherosclerosis or macrophage polarization in mice. J Lipid Res. 2013; 54:806–815. doi: 10.1194/jlr.M034157 23288947 10.1194/jlr.M034157 PMC3617954

[12] Hanna RN Shaked I Hubbeling HG Punt JA Wu R Herrley E Zaugg C Pei H Geissmann F Ley K NR4A1 (Nur77) deletion polarizes macrophages toward an inflammatory phenotype and increases atherosclerosis. Circ Res. 2012; 110:416–427. doi: 10.1161/CIRCRESAHA.111.253377 22194622 10.1161/CIRCRESAHA.111.253377 PMC3309661

[13] Tanigaki K Han H Yamamoto N Tashiro K Ikegawa M Kuroda K Suzuki A Nakano T Honjo T Notch-RBP-J signaling is involved in cell fate determination of marginal zone B cells. Nat Immunol. 2002; 3:443–450. doi: 10.1038/ni793 11967543 10.1038/ni793

[14] Binder CJ Hörkkö S Dewan A Chang MK Kieu EP Goodyear CS Shaw PX Palinski W Witztum JL Silverman GJ Pneumococcal vaccination decreases atherosclerotic lesion formation: molecular mimicry between Streptococcus pneumoniae and oxidized LDL. Nat Med. 2003; 9:736–743. doi: 10.1038/nm876 12740573 10.1038/nm876

[15] Chou MY Fogelstrand L Hartvigsen K Hansen LF Woelkers D Shaw PX Choi J Perkmann T Bäckhed F Miller YI Oxidation-specific epitopes are dominant targets of innate natural antibodies in mice and humans. J Clin Invest. 2009; 119:1335–1349. doi: 10.1172/JCI36800 19363291 10.1172/JCI36800 PMC2673862

[16] Rinninger F Heine M Singaraja R Hayden M Brundert M Ramakrishnan R Heeren J High density lipoprotein metabolism in low density lipoprotein receptor-deficient mice. J Lipid Res. 2014; 55:1914–1924. doi: 10.1194/jlr.M048819 24954421 10.1194/jlr.M048819 PMC4617360

[17] Tsiantoulas D Kiss M Bartolini-Gritti B Bergthaler A Mallat Z Jumaa H Binder CJ Secreted IgM deficiency leads to increased BCR signaling that results in abnormal splenic B cell development. Sci Rep. 2017; 7:3540 doi: 10.1038/s41598-017-03688-8 28615655 10.1038/s41598-017-03688-8 PMC5471202

[18] Karki K Wright GA Mohankumar K Jin UH Zhang XH Safe S A bis-indole-derived NR4A1 antagonist induces PD-L1 degradation and enhances anti-tumor immunity. Cancer Res. 2020; 80:1011–1023. doi: 10.1158/0008-5472.CAN-19-2314 31911554 10.1158/0008-5472.CAN-19-2314 PMC7056589

[19] Gotsman I Grabie N Dacosta R Sukhova G Sharpe A Lichtman AH Proatherogenic immune responses are regulated by the PD-1/PD-L pathway in mice. J Clin Invest. 2007; 117:2974–2982. doi: 10.1172/JCI31344 17853943 10.1172/JCI31344 PMC1974866

[20] Doyon-Laliberté K Chagnon-Choquet J Byrns M Aranguren M Memmi M Chrobak P Stagg J Poudrier J Roger M NR4A expression by human marginal zone B-cells. Antibodies (Basel). 2019; 8:50 doi: 10.3390/antib8040050 10.3390/antib8040050 PMC6963983 31614541

[21] Hedrick E Li X Cheng Y Lacey A Mohankumar K Zarei M Safe S Potent inhibition of breast cancer by bis-indole-derived nuclear receptor 4A1 (NR4A1) antagonists. Breast Cancer Res Treat. 2019; 177:29–40. doi: 10.1007/s10549-019-05279-9 31119568 10.1007/s10549-019-05279-9 PMC6681651

[22] Park K Nowyhed H Hedrick C Nr4a1 (Nur77) regulates B cell survival and activation. (P1453). J Immunol. 2013; 190 1 suppl 174.9 LP–174.9. http://www.jimmunol.org/content/190/1_Supplement/174.9.abstract.23197258

[23] Tan C Hiwa R Mueller JL Vykunta V Hibiya K Noviski M Huizar J Brooks J Garcia J Heyn C “A negative feedback loop mediated by the NR4A family of nuclear hormone receptors restrains expansion of B cells that receive signal one in the absence of signal two.” bioRxiv. 2020 doi: 10.1101/2020.03.31.017434

[24] Echeverri Tirado LC Yassin LM B cells interactions in lipid immune responses: implications in atherosclerotic disease. Lipids Health Dis. 2017; 16:30 doi: 10.1186/s12944-016-0390-5 28166809 10.1186/s12944-016-0390-5 PMC5295187

[25] Douna H Amersfoort J Schaftenaar FH Kroon S van Puijvelde GHM Kuiper J Foks AC Bidirectional effects of IL-10+ regulatory B cells in Ldlr-/- mice. Atherosclerosis. 2019; 280:118–125. doi: 10.1016/j.atherosclerosis.2018.11.019 30500604 10.1016/j.atherosclerosis.2018.11.019

[26] Jackson SW Scharping NE Jacobs HM Wang S Chait A Rawlings DJ Cutting edge: BAFF overexpression reduces atherosclerosis via TACI-dependent B cell activation. J Immunol. 2016; 197:4529–4534. doi: 10.4049/jimmunol.1601198 27837104 10.4049/jimmunol.1601198 PMC5147509

