# Supplementary material for: NR4A1 Deletion in Marginal Zone B Cells Exacerbates Atherosclerosis in Mice—Brief Report
Source: Arterioscler Thromb Vasc Biol. 2020 Sep 10;40(11):2598–604. doi: 10.1161/ATVBAHA.120.314607 (PMC7571845; doi:10.1161/ATVBAHA.120.314607)
Supplement: Supplementary file 1 [file atv-40-2598-s001.pdf]

SUPLLEMENTAL MATERIAL

NR4A1 deletion in marginal zone B cells exacerbates atherosclerosis in mice

Meritxell Nus, PhD[1,2]*, Gemma Basatemur, PhD[1], Maria Galan, PhD[2,3], Laia Cros-Brunsó, BSc[1], Tian X Zhao, MD[1], Leanne Masters BSc[1], James Harrison, BSc[1], Nichola Figg, BSc[1], Dimitrios Tsiantoulas, PhD[4], Frederic Geissmann, MD PhD[5], Christoph J Binder, MD PhD[4], Andrew P Sage, PhD[1], Ziad Mallat, MD PhD[1,6]

[1]Division of Cardiovascular Medicine, University of Cambridge, UK; [2]CIBER de Enfermedades Cardiovasculares (CIBERCV), España; [3]Biomedical Research Institute, Hospital de la Santa Creu i Sant Pau, Barcelona, Spain; [4]Department of Laboratory Medicine, Medical University of Vienna, Austria; [5]Memorial Sloan Kettering Cancer Centre, New York 10065, USA; [6]Institut National de la Santé et de la Recherche Médicale (Inserm), Unit 970, Paris Cardiovascular Research Center, Paris, France.

Short tittle: B cells Nr4a1 in atherosclerosis

***Corresponding author:** Dr Meritxell Nus, Dept. of Medicine, Level 5 Box 157, Addenbrooke´s Hospital, CB2 0QQ Cambridge, United Kingdom. Email: mn421@cam.ac.uk

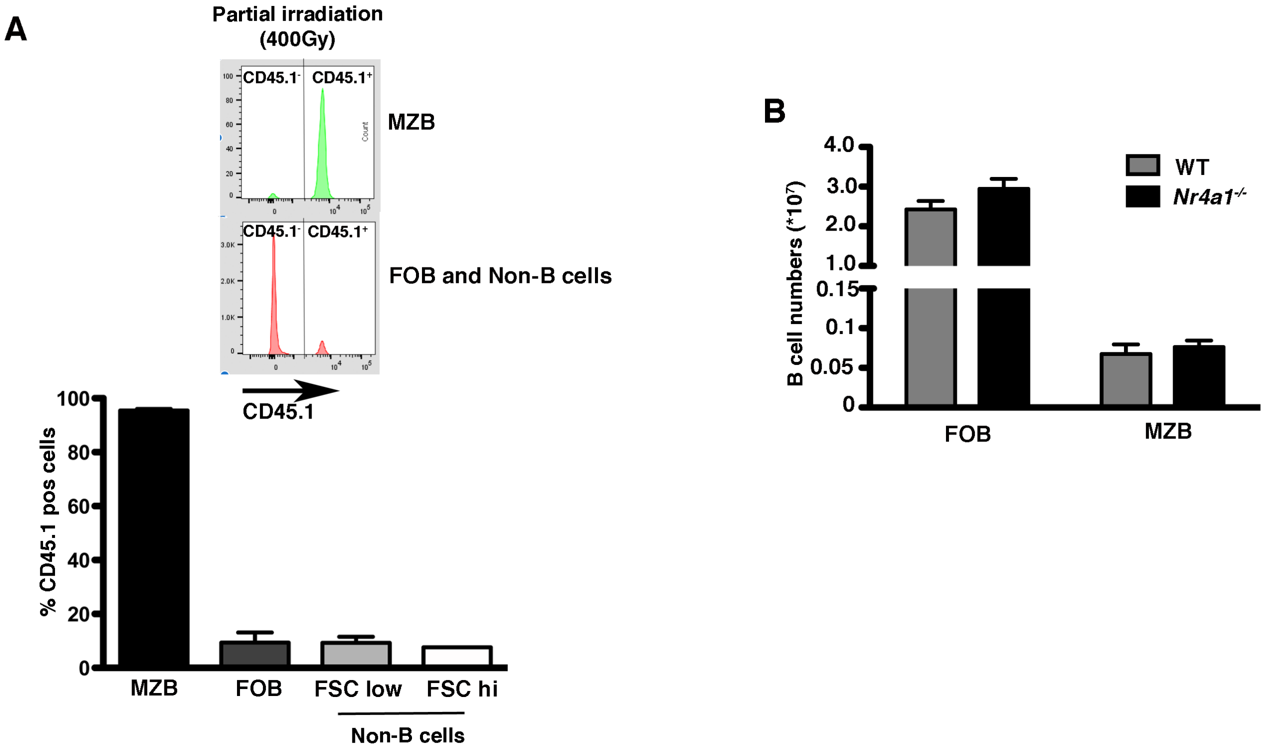

Supplementary Figure I. Characterization of the partial irradiation model. (A) Graph showing percentage of CD45.1^{+} MZB, FOB and Non-B cells (including FSC^{low}: other lymphocytes and FSC^{hi}: myeloid) cells in CD45.2 *Ldlr*$^{-/-}$*; Cd79a*$^{Cre/+}$*; RBP*$^{flox/flox}$ mice injected with CD45.1 BM donor cells after partial irradiation with a single dose of 4 Gy. There were 3 experiments with 3 mice per group. (B) Total numbers of B2 cell subsets from males and females *Ldlr*$^{-/-}$*; Cd79a*$^{Cre/+}$*; Rbpjk*$^{flox/flox}$ mice partially irradiated and injected with WT (for reconstitution with WT MZB cells) or *Nr4a1*$^{-/-}$ (for reconstitution with *Nr4a1*$^{-/-}$ MZB cells) BM and fed a HF/HC diet for 8 weeks. Two-way ANOVA.

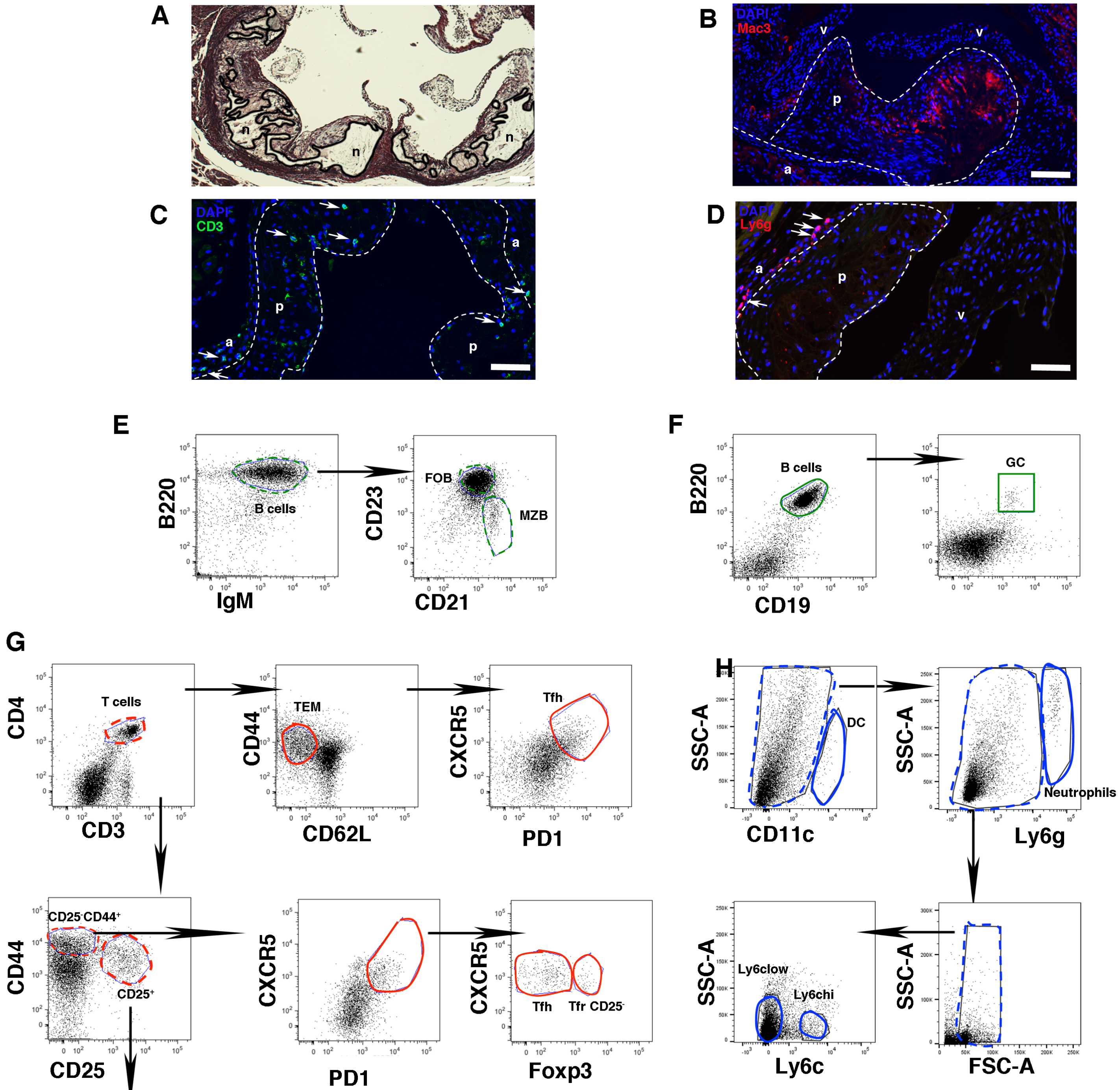

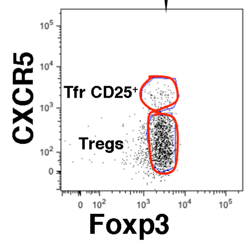

Supplementary Figure II. Representative immunofluorescence images and flow cytometry plots for immune cell subsets. Representative images of aortic root sections from mice used in this paper showing: (A) Necrotic core (n); (B) Mac3 staining (red) for macrophages in atherosclerotic plaques, and (C) $CD3^{+}$ (green) and (D) $Ly6G^{+}$ cells (red) in atherosclerotic plaques and in adventitia. Original magnification 10x (A) and 20x (B-D). Scale bar 50 μm. Representative plots and gating strategy for (E) B cell subsets: B cells (IgM^{+} $B220^{+}$); FOB (IgM^{+} $B220^{+}CD23^{+}CD21^{low}$) and MZB cells ($IgM^{+}$ $B220^{+}CD23^{low}CD21^{hi}$); (F) GC B cells ($CD19^{+}$ $B220^{+}CD95^{hi}Gl7^{hi}$); (G) CD4 T cells ($CD3^{+}CD4^{+}$); TEM ($CD3^{+}$ $CD4^{+}CD44^{hi}CD62L^{-}$); Tfh ($CD3^{+}$ $CD4^{+}CD44^{hi}CXCR5^{+}PD1^{+}$); Tregs ($CD3^{+}$ $CD4^{+}CD44^{+}CD25^{+}Foxp3^{+}CXCR5^{-}$ $PD1^{-}$); $TfrCD25^{+}$ ($CD3^{+}CD4^{+}CD44^{+}CD25^{+}Foxp3^{+}$ $CXCR5^{+}PD1^{+}$); $TfrCD25^{-}$ ($CD3^{+}CD4^{+}CD44^{hi}CD25^{-}Foxp3^{+}$ $CXCR5^{+}PD1^{+}$); H) DC ($CD11c^{+}$); neutrophils ($Ly6G^{+}$); Monocytes ($CD11c^{-}Ly6G^{-}Ly6C^{hi/low}$).

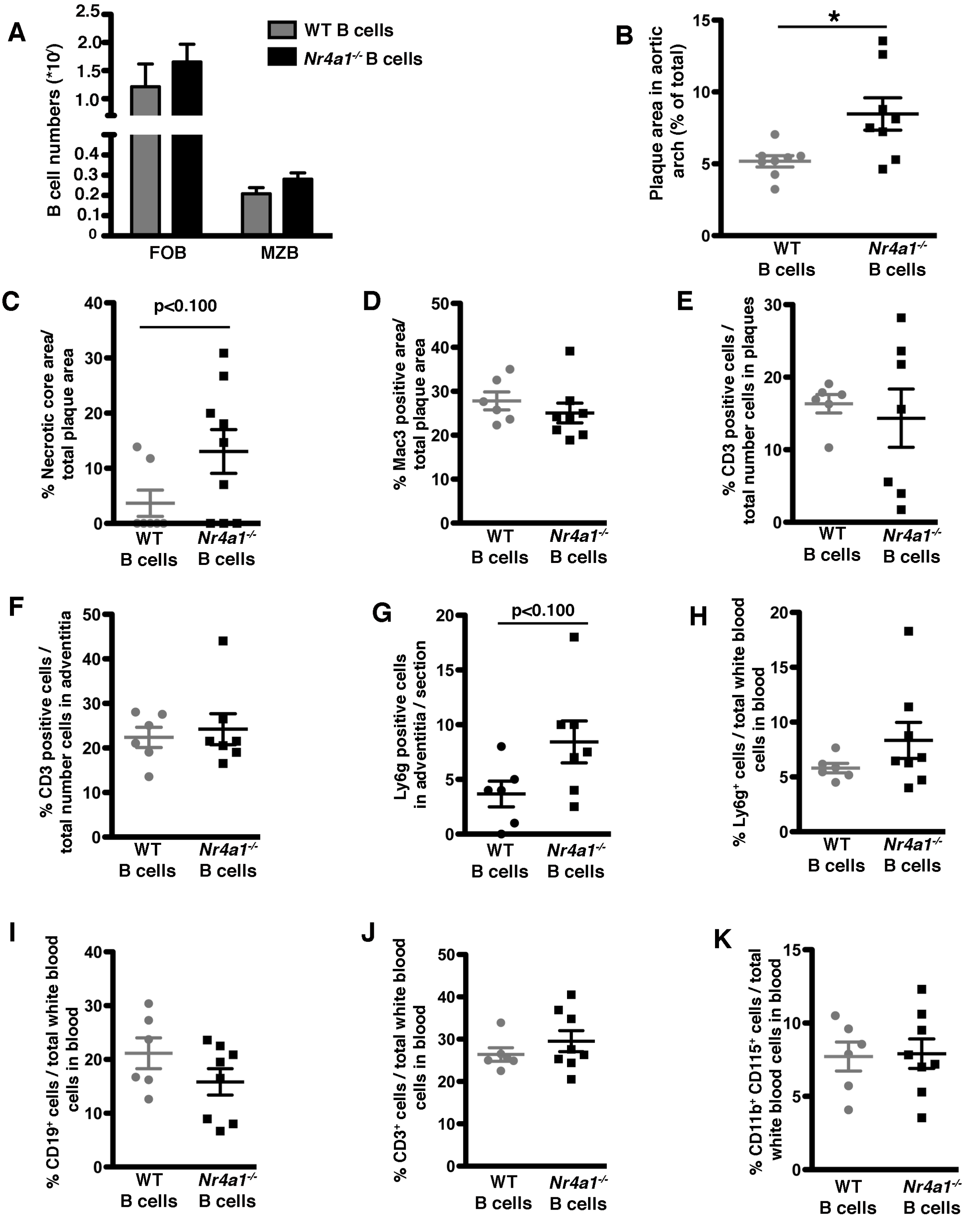

Supplementary Figure III. Further characterization of atherosclerotic plaques and blood from *Ldlr*$^{-/-}$ reconstituted with WT or *Nr4a1*$^{-/-}$ B cells. *Ldlr*$^{-/-}$ male mice were transplanted with a mixed chimera containing 80% *μMT* + 20% WT (for reconstitution with WT B cells) or 20% *Nr4a1*$^{-/-}$ (for reconstitution with *Nr4a1*$^{-/-}$ B cells) BM and fed a HF/HC diet for 8 weeks (A-K). (A) Numbers of splenic MZB and FOB cells. (B) Quantification of Oil red O en face staining of aortic arches. Quantification of (C) necrotic core area, (D) Mac3 and (E) CD3 positive staining in atherosclerotic plaques of aortic roots. Quantification of (F) CD3 positive cells and (G) neutrophils (Ly6G positive cells) in adventitia of aortic root sections. For (C-G) 3 representative sections were analyzed for each mouse with n=6-8 per group. Percentage of blood (H) neutrophils, (I) B cells, (J) T cells and (K) monocytes. For (B-K) each symbol represents one mouse and horizontal bars are group mean ± s.e.m. Two-tailed unpaired Student t-test *p<0.05.

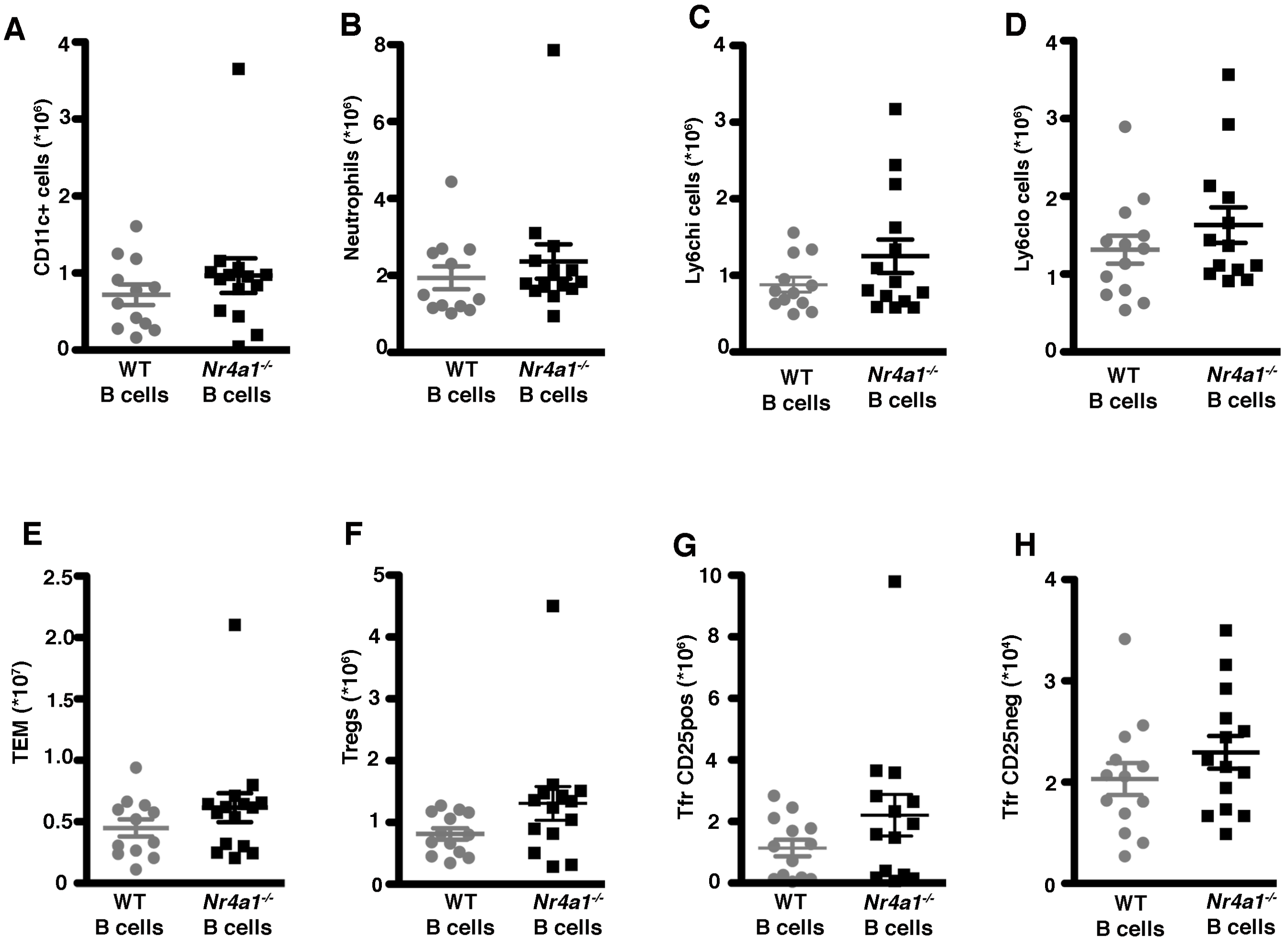

Supplementary Figure IV. Further splenic immunophenotyping of *Ldlr*$^{-/-}$ reconstituted with WT or *Nr4a1*$^{-/-}$ B cells. *Ldlr*$^{-/-}$ male mice were transplanted with a mixed chimera containing 80% *µMT* + 20% WT (for reconstitution with WT B cells) or 20% *Nr4a1*$^{-/-}$ (for reconstitution with *Nr4a1*$^{-/-}$ B cells) BM and fed a HF/HC diet for 8 weeks (A-H). Total numbers of splenic (A) CD11c positive (dendritic cells); (B) Ly6G^{+} (neutrophils); (C-D) Ly6C$^{hi/low}$ (monocytes) ; (E) T effector memory (TEM) cells; (F) T regulatory Foxp3^{+} cells; (G) T folicular regulatory CD25^{+} cells and (H) T folicular regulatory CD25^{-} cells. Each symbol represents one mouse; horizontal bars are group mean ± s.e.m.. Two-tailed unpaired Student t-test.

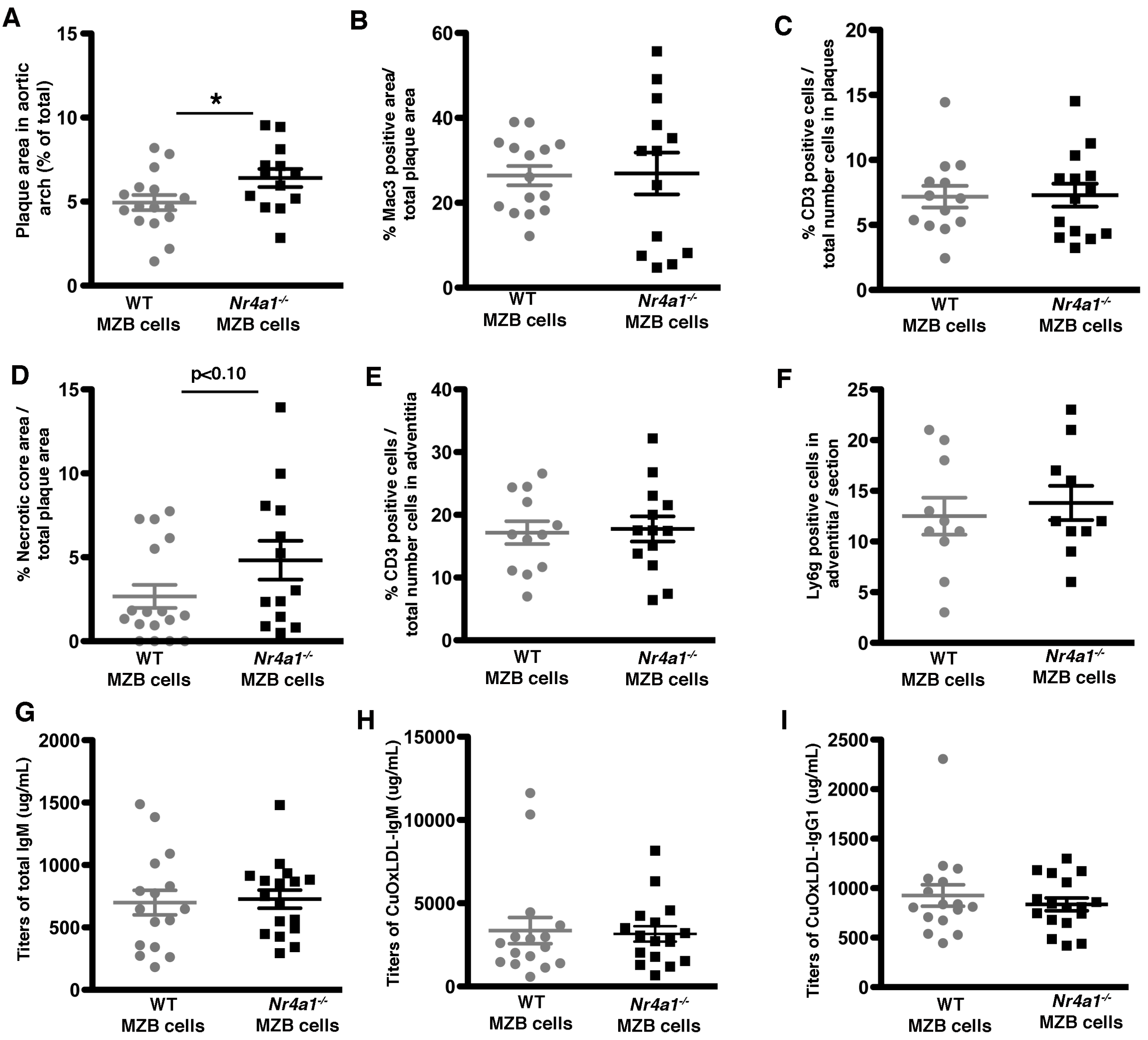

Supplementary Figure V. Further characterization of atherosclerotic plaques of *Ldlr*$^{-/-}$; *Cd79a*$^{Cre/+}$; *RBP*$^{flox/flox}$ mice reconstituted with WT or *Nr4a1*$^{-/-}$ MZB cells. *Ldlr*$^{-/-}$; *Cd79a*$^{Cre/+}$; *Rbpjk*$^{flox/flox}$ male and female mice were partially irradiated and injected with WT (for reconstitution with WT MZB cells) or *Nr4a1*$^{-/-}$ (for reconstitution with *Nr4a1*$^{-/-}$ MZB cells) BM and fed a HF/HC diet for 8 weeks. (A) Quantification of Oil red O en face staining of aortic arches. Quantification of (B) Mac3 and (C) CD3 positive staining and (D) necrotic core area, in atherosclerotic plaques of aortic roots. Quantification of (E) CD3 positive cells and (F) neutrophils (Ly6G^{+} cells) in adventitia of aortic root sections. For (B-F) 3 representative sections were analyzed for each mouse with n=10-15 per group. Total plasma (G) IgM, (H) CuOxLDL specific IgM and (I) CuOxLDL specific IgG1 antibody titres. Each symbol represents one mouse and horizontal bars are group mean ± s.e.m. Two-tailed unpaired Student t-test *$p<0.05$.

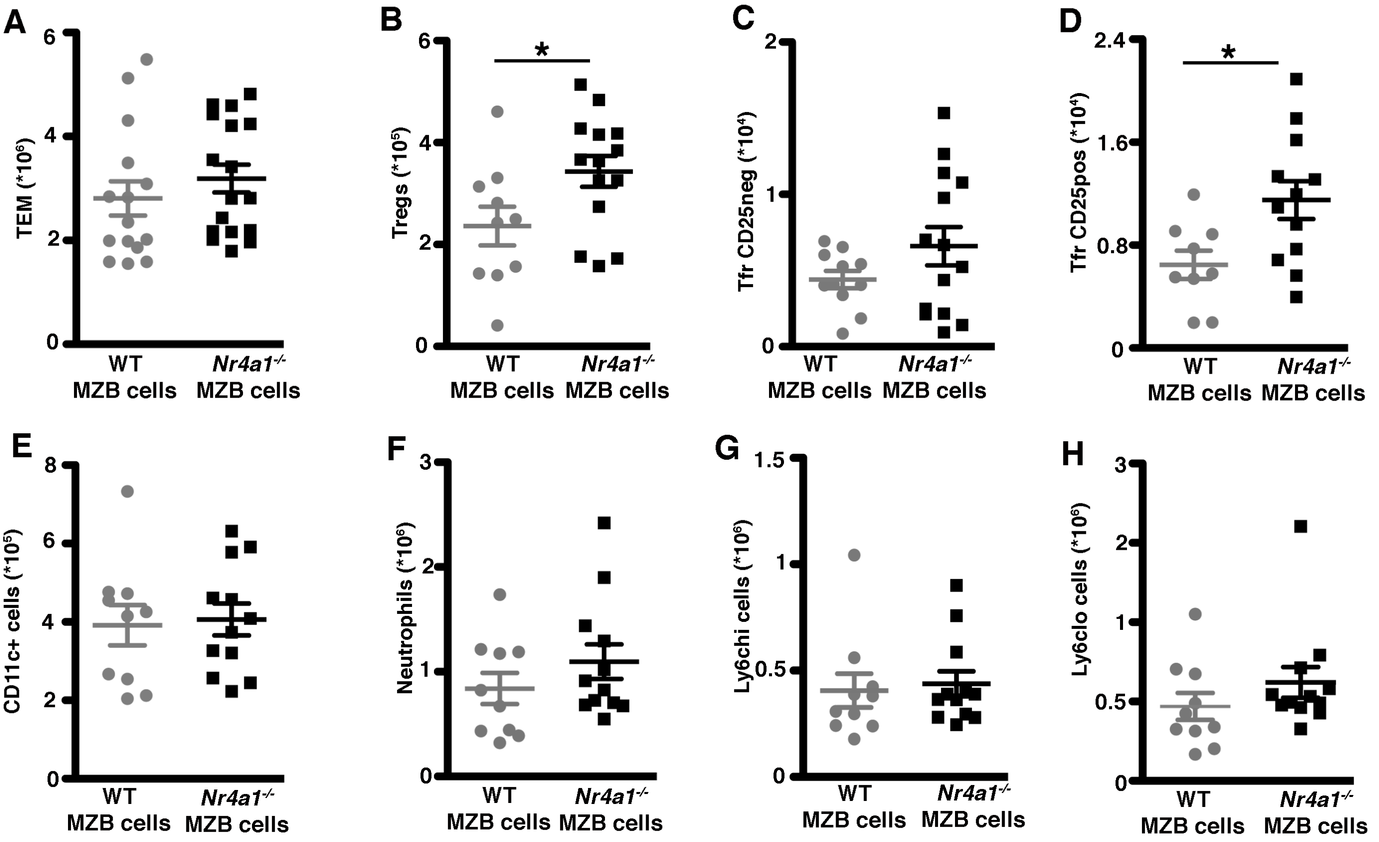

Supplementary Figure VI. Further splenic immunophenotyping of *Ldlr$^{-/-}$; Cd79a$^{Cre/+}$; RBP$^{flox/flox}$* mice reconstituted with WT or *Nr4a1$^{-/-}$* MZB cells. *Ldlr$^{-/-}$; Cd79a$^{Cre/+}$; Rbpjk$^{flox/flox}$* male and female mice were partially irradiated and injected with WT (for reconstitution with WT MZB cells) or *Nr4a1$^{-/-}$* (for reconstitution with *Nr4a1$^{-/-}$* MZB cells) BM and fed a consumption of a HF/HC diet for 8 weeks. Total numbers of splenic (A) T effector memory (TEM) cells; (B) T regulatory Foxp3^{+} cells; (C) T follicular regulatory CD25^{+} cells and (D) T follicular regulatory CD25^{-} cells; (E) CD11c^{+} (dendritic cells); (F) Ly6G^{+} (neutrophils); (G-H) Ly6C$^{hi/low}$ (monocytes). Each symbol represents one mouse; horizontal bars are group mean ± s.e.m. Two-tailed unpaired Student t-test. *p<0.05.

*

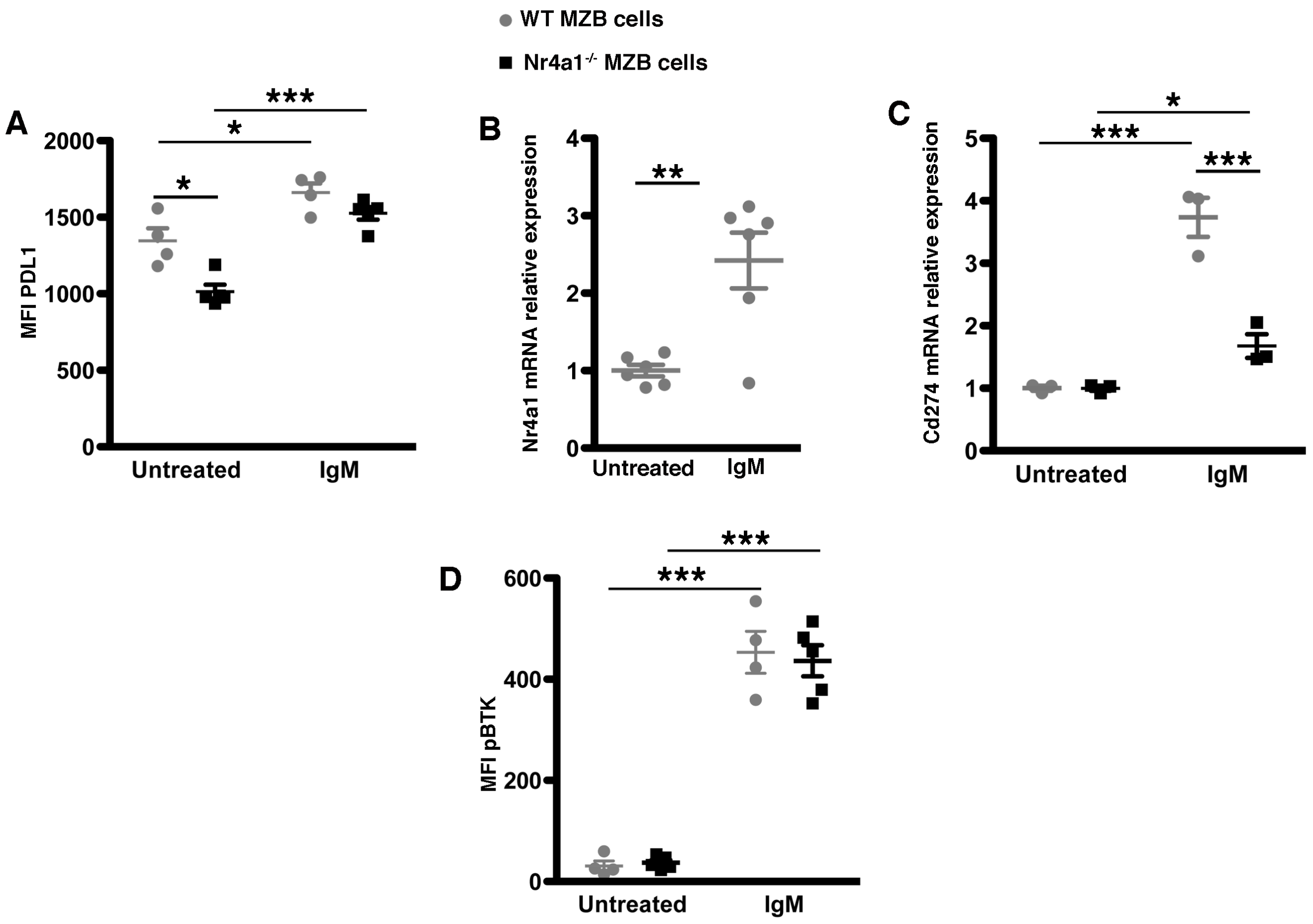

Supplementary Figure VII. NR4A1 modulates PDL1 expression in MZB cells in a BCR-independent manner. MZB cells were sorted from WT or *Nr4a1*$^{-/-}$ mice and were treated *in vitro* with anti-IgM for (A) 6 hours prior to assessment of PDL1 protein expression by flow cytometry, or for 3 hours prior to assessment of (B) *Nr4a1* and (C) *Cd274* expression by qRT-PCR, or (D) for 30 min prior to assessment of Btk phosphorylation (pBtk) by flow cytometry. (n=4-6 biological replicates per group). Horizontal bars are mean ± s.e.m. Two-tailed unpaired Student t-test and two-way ANOVA followed by Bonferroni´s *post-hoc* analysis. * $p<0.05$; ** $p<0.01$ and *** $p<0.001$.

Major Resources Table

In order to allow validation and replication of experiments, all essential research materials listed in the Methods should be included in the Major Resources Table below. Authors are encouraged to use public repositories for protocols, data, code, and other materials and provide persistent identifiers and/or links to repositories when available. Authors may add or delete rows as needed.

Animals (in vivo studies)

Species	Vendor or Source	Background Strain	Sex	Persistent ID / URL
Mice WT	Charles River	C57Bl/6J	M-F	https://www.criver.com/products-services/find-model/c57bl6-mouse?region=3671

Genetically Modified Animals

	Species	Vendor or Source	Background Strain	Other Information	Persistent ID / URL
**Parent - Male**	LDLr-/-	Jackson Labs	C57Bl/6J		MGI: 1857212
**Parent - Female**	LDLr-/-	Jackson Labs	C57Bl/6J		MGI: 1857212
**Parent - Male**	RBPf/f	T. Honjo	C57Bl/6J		MGI:3583755
**Parent - Female**	RBPf/f	T. Honjo	C57Bl/6J		MGI:3583755
**Parent - Male**	CD79aCre/+	Jackson Labs	C57Bl/6J		MGI:3687451
**Parent - Female**	CD79a+/+	Jackson Labs	C57Bl/6J		MGI:3687451
**Parent - Male**	Nr4a1-/-	Jackson Labs	C57Bl/6J		MGI:1352454
**Parent - Female**	Nr4a1-/-	Jackson Labs	C57Bl/6J		MGI:1352454

Antibodies

Target antigen	Vendor or Source	Catalog #	Working concentration	Lot # (preferred but not required)	Persistent ID / URL
B220	eBioscience	103243	1ug/mL		https://www.biolegend.com/en-us/products/brilliant-violet-605-anti-mouse-human-cd45r-b220-antibody-7870
IgM	eBio	48-5890-82	1ug/mL		https://www.thermofisher.com/antibody/product/IgM-Antibody-clone-eB121-15F9-Monoclonal/48-5890-82
MHC-II	Biolegend	107620	1ug/mL		https://www.biolegend.com/en-us/products/pacific-blue-anti-mouse-i-a-i-e-antibody-3136
CD23	eBioscience	12-0232-82	1ug/mL		https://www.thermofisher.com/antibody/product/CD23-Antibody-clone-B3B4-Monoclonal/12-0232-82
CD21	eBioscience	123418	1ug/mL		https://www.thermofisher.com/antibody/product/CD21-Antibody-clone-HB5-Monoclonal/11-0219-42

DOI [to be added]

CD69	Biolegend	104508	2ug/mL		https://www.biolegend.com/en-us/products/pe-anti-mouse-cd69-antibody-265
CD44	Biolegend	103047	1ug/mL		https://www.biolegend.com/en-us/products/brilliant-violet-605-anti-mouse-human-cd44-antibody-8807
CD4	Biolegend	100536	1ug/mL		https://www.biolegend.com/en-us/products/alexa-fluor-700-anti-mouse-cd4-antibody-3386
CD3	Biolegend	100321	1ug/mL		https://www.biolegend.com/en-us/products/alexa-fluor-488-anti-mouse-cd3epsilon-antibody-2676
CD62L	Biolegend	104424	1ug/mL		https://www.biolegend.com/en-us/products/pacific-blue-anti-mouse-cd62l-antibody-3117
CD95	BD	557653	2ug/mL		https://www.bdbiosciences.com/eu/applications/research/t-cell-immunology/regulatory-t-cells/surface-markers/mouse/pe-cy7-hamster-anti-mouse-cd95-jo2/p/557653
GL7	Biolegend	144607	2ug/mL		https://www.biolegend.com/en-us/products/pe-anti-mouse-human-gl7-antigen-t-and-b-cell-activation-marker-antibody-9122
CD19	BD	553785	2ug/mL		https://www.bdbiosciences.com/eu/applications/research/stem-cell-research/hematopoietic-stem-cell-markers/mouse/negative-markers/fitc-rat-anti-mouse-cd19-1d3/p/553785
CD138	Biolegend	142505	2ug/mL		https://www.biolegend.com/en-us/products/apc-anti-mouse-cd138-syndecan-1-antibody-7572
CXCR5	Biolegend	145503	2ug/mL		https://www.biolegend.com/en-us/products/pe-anti-mouse-cd185-cxcr5-antibody-8455
ICOS	eBioscience	17-9949-82	2ug/mL		https://www.thermofisher.com/antibody/product/CD278-ICOS-Antibody-clone-C398-4A-Monoclonal/17-9949-82
PD1	Biolegend	135241	2ug/mL		https://www.biolegend.com/en-us/products/brilliant-violet-510-anti-mouse-cd279-pd-1-antibody-14923
pBtk	eBioscience	17-9015-42	8.3 ug/mL		https://www.thermofisher.com/antibody/product/Phospho-BTK-ITK-Tyr551-Tyr511-Antibody-clone-M4G3LN-Monoclonal/17-9015-42
PDL1	eBioscience	46-5982-82	2ug/mL		https://www.thermofisher.com/antibody/product/CD274-PD-L1-B7-H1-Antibody-clone-MIH5-Monoclonal/46-5982-82

DNA/cDNA Clones N/A

Cultured Cells N/A

Data & Code Availability N/A

Other N/A

DOI [to be added]
