# Supplementary figures and images for: NR4A1 Deletion in Marginal Zone B Cells Exacerbates Atherosclerosis in Mice—Brief Report
Source: Arterioscler Thromb Vasc Biol. 2020 Sep 10;40(11):2598–604. doi: 10.1161/ATVBAHA.120.314607 (PMC7571845; doi:10.1161/ATVBAHA.120.314607)

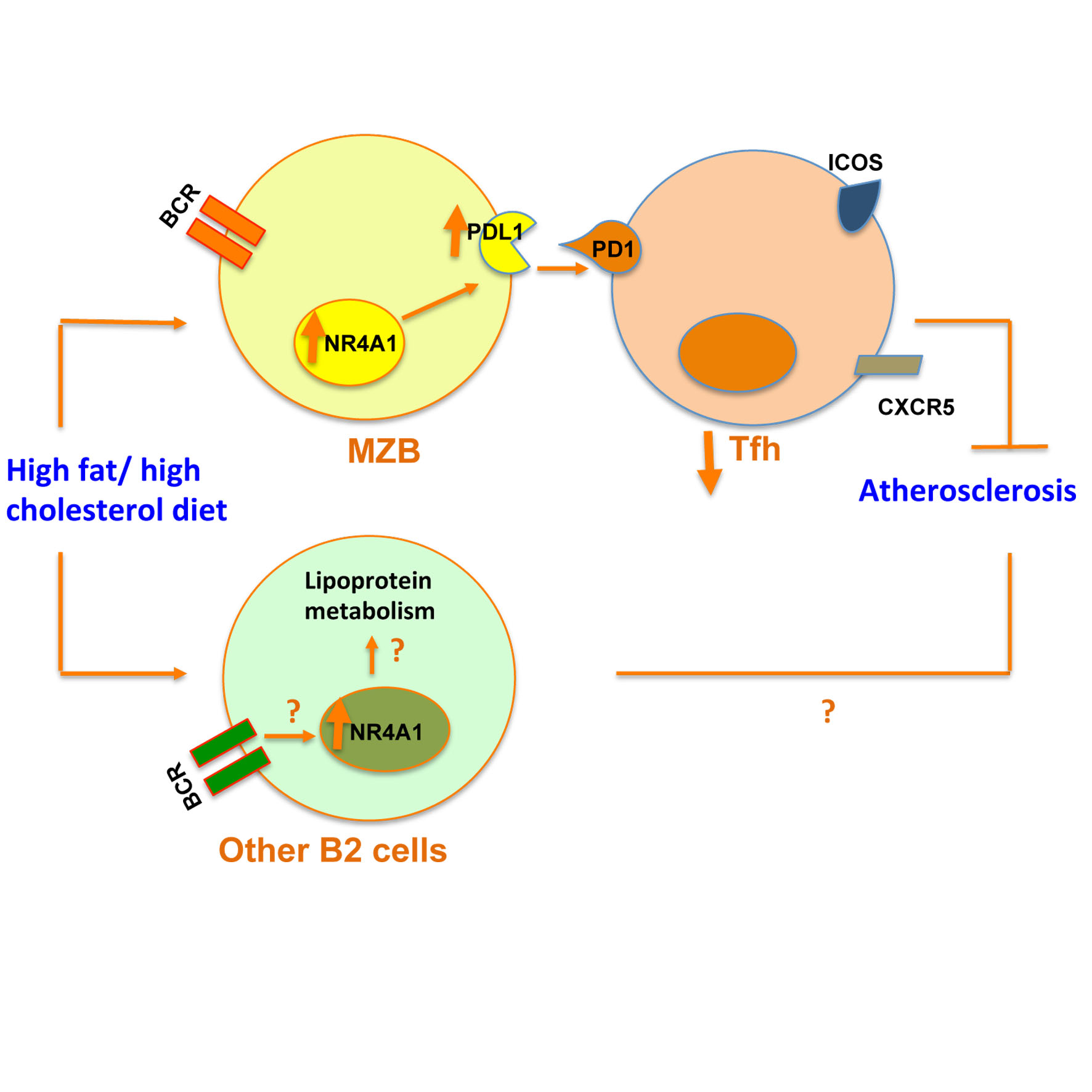

Supplement: Supplementary file 2 [file atv-40-2598-s002.jpg]
